# Intestinal and neurological involvement in Behcet disease: a clinical case

**DOI:** 10.1186/s13052-017-0350-3

**Published:** 2017-04-07

**Authors:** Romina Gallizzi, Dominique De Vivo, Simona Valenti, Caterina Pidone, Carmelo Romeo, Rosario Caruso, Claudio Romano

**Affiliations:** 1 Department of Human Pathology in Adulthood and Childhood “G. Barresi”, Unit of Pediatrics, University of Messina, Messina, Italy; 2 Department of Human Pathology in Adulthood and Childhood “G. Barresi”, Unit of Pediatric Surgery, University of Messina, Messina, Italy; 3 Department of Human Pathology in Adulthood and Childhood “G. Barresi”, Section of Anatomic Pathology, University of Messina, Messina, Italy

## Abstract

**Background:**

Behcet’s disease (BD) is a chronic immune-mediated, inflammatory disorder which may affect a number of different systems (oral and genital mucosa, eyes, skin, vascular district, joints, gastrointestinal tract and nervous system). Neurological manifestations are present in 5–10%, and gastrointestinal tract involvement in 10–15% of cases. The simultaneous involvement of two systems, neurological and gastrointestinal tract, is very rare and represents the aim of our case report.

**Case presentation:**

We describe a case of a 12-year-old girl with neurological (endocranial hypertension, papilledema, retinal vasculitis) and gastrointestinal tract (terminal ileum and cecum inflammation) involvement and with a history of recurrent oral aphthosis; therefore, according to both International Criteria for Behcet’s Disease (ICBD) and Paediatric Behcet’s Disease criteria (PEDBD) the diagnosis of BD was confirmed.

**Conclusions:**

This case report is one of the few described in literature with simultaneous involvement of the two systems, neurological and gastrointestinal tract, in paediatric BD. The diagnosis is really difficult because there is no specific diagnostic test. We think that our clinical case should help clinicians to suspect a BD with an unusual onset.

## Background

Behçet disease (BD) was first described by the Turkish dermatologist Hulusi Behçet in 1937 as a syndrome with oral and genital ulcerations and ocular inflammation [1, 2]. It is defined as a rare multi-systemic inflammatory disease with unknown etiology and chronic recurrent pattern, characterized by oral and genital aphthosis/ulcers with ocular, skin, articular, vascular, gastrointestinal and/or central nervous system lesions. Furthermore, it has many clinical characteristics that are similar to inflammatory bowel diseases (IBD). BD is included both in vasculitis and auto-inflammatory disease classifications [3]. It is considered a vasculitis with involvement of vessels of all kinds and sizes [4] and it is defined as a multifactorial auto-inflammatory syndrome [5]. The highest prevalence of BD has been reported in Japan, Iran, Turkey (Silk Route), more rarely occurring in Western countries. Usually, BD onset is found in young adults, around 20–35 years, with no difference between sexes, but a worse disease course in the male population. BD is rare in paediatric age and the prevalence is unknown. It is difficult to make diagnosis under 16 years due to the heterogeneous clinical picture, such as oral and genital ulcers, erythema nodosum, superficial thrombophlebitis, acne and arthritis [6, 7]. Factors that contribute to the pathogenesis of BD include the host’s genetic profile and immune system, and environmental factors such as the gut microbiota. BD has an important genetic component, and thus the frequency of familial cases is around 10 to 50% [8]. HLA-B51 is associated with BD, being predominant in affected males with a higher prevalence of genital ulcers, ocular and skin manifestations and decreased gastrointestinal involvement [9]. Diagnosis of BD is based on clinical criteria. The most widely used diagnostic criteria for adult onset disease are from the International Behcet’s Study Group (ISG). In 2014, new criteria for BD diagnosis were proposed, called ICBD, that include two additional clinical criteria, neurological and vascular involvement, permitting diagnosis even without the presence of oral aphthous lesions which were considered mandatory in the previous ISG classification. An international expert consensus group, the pediatric BD (PEDBD), has recently proposed a new set of criteria for the classification of BD in children [10]. PEDBD include the following features: recurrent oral aphtosis (at least 3 attacks/year), genital ulceration (typically with scar), skin involvement (necrotic folliculitis, acneiform lesions, erythema nodosum), ocular involvement (anterior or posterior uveitis, retinal vasculitis), neurological signs (with the exception of isolated headaches), vascular signs (venous thrombosis, arterial thrombosis, arterial aneurysm). All the clinical symptoms have the same importance (1 point) and three of them are required to classify a patient as having pediatric BD. Unlike the ISG and ICBD criteria, in PEDBD the pathergy test is not included. In children the symptoms are few to apply any classification. Because of the exiguity of clinical manifestations in the pediatric population, in most case the diagnosis is based on the physician’s experience. A substantial difference between the ISG and the PEDBD/ICBD criteria are that oral aphthosis is the mandatory criterion, while in the other two classification, it is possible to make diagnosis of BD even without the presence of oral aphtosis. The treatment of BD is difficult and is based on disease activity and severity including different medical therapies (corticosteroids, colchicine, immunosuppressive and biological drugs).

## Case presentation

**Fig. 1:**
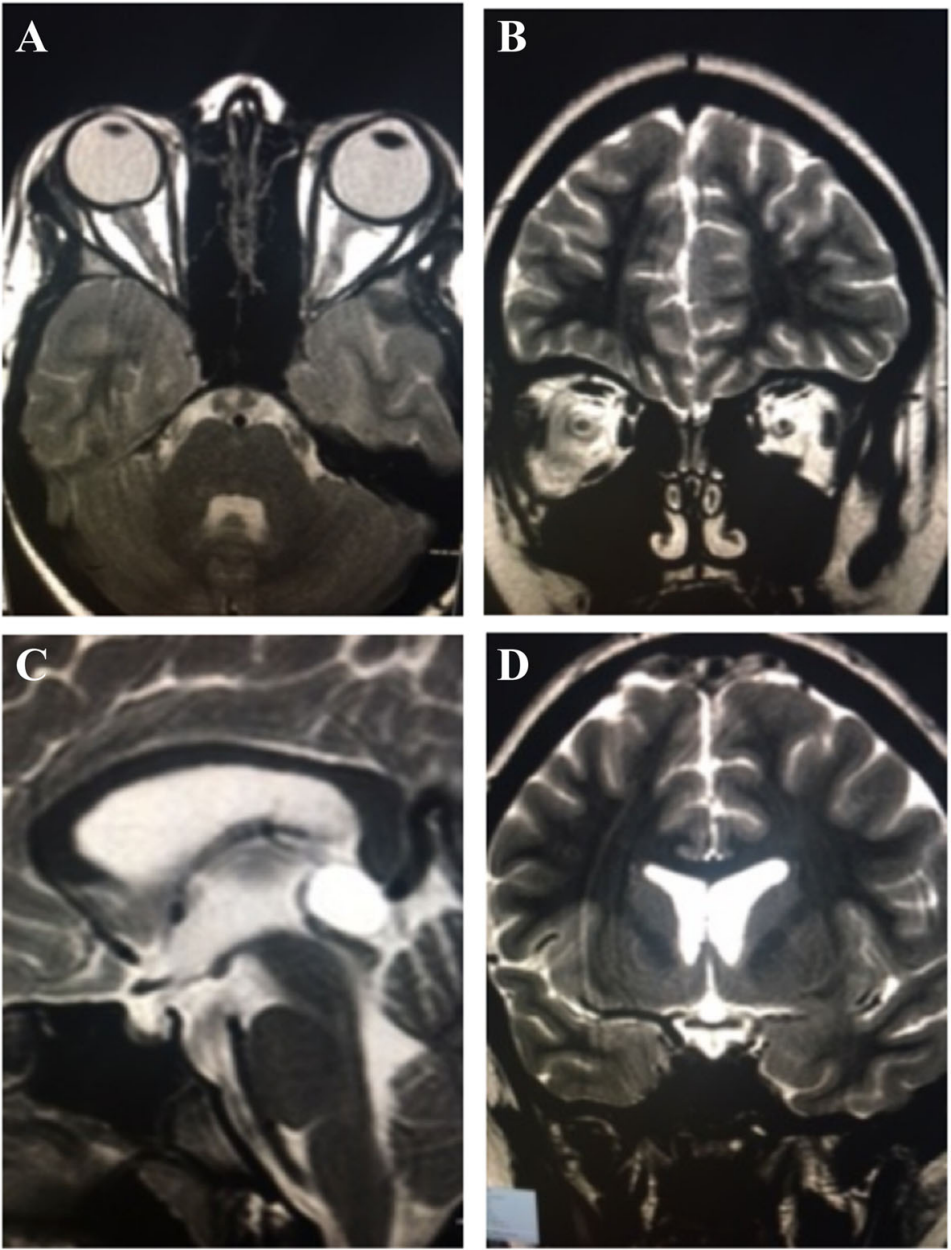
Brain Magnetic Resonance Imaging MRI TSE sequences of the patient showing mild inversion of the optic papilla on the optic disk on both sides, with prominent subaracnoid space around the optic nerves (**a**-**b**), and a partially empty sella turcica (**c**-**d**)

**Fig. 2:**
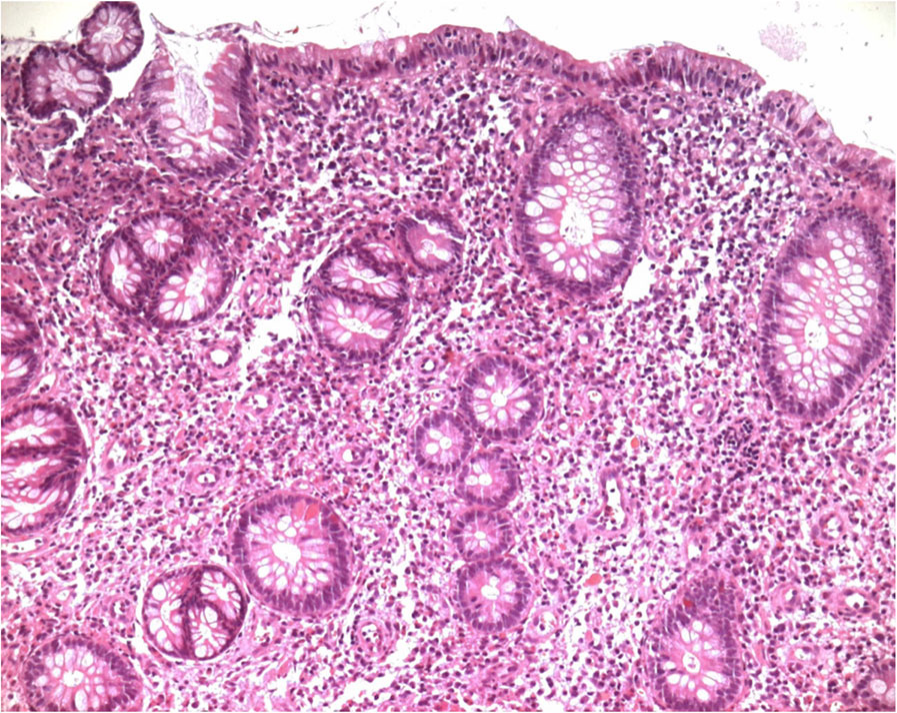
Histology from the ileocecal valve showing inflammatory cell infiltration and loss of glands Hematoxylin-eosin x 100

**Fig. 3:**
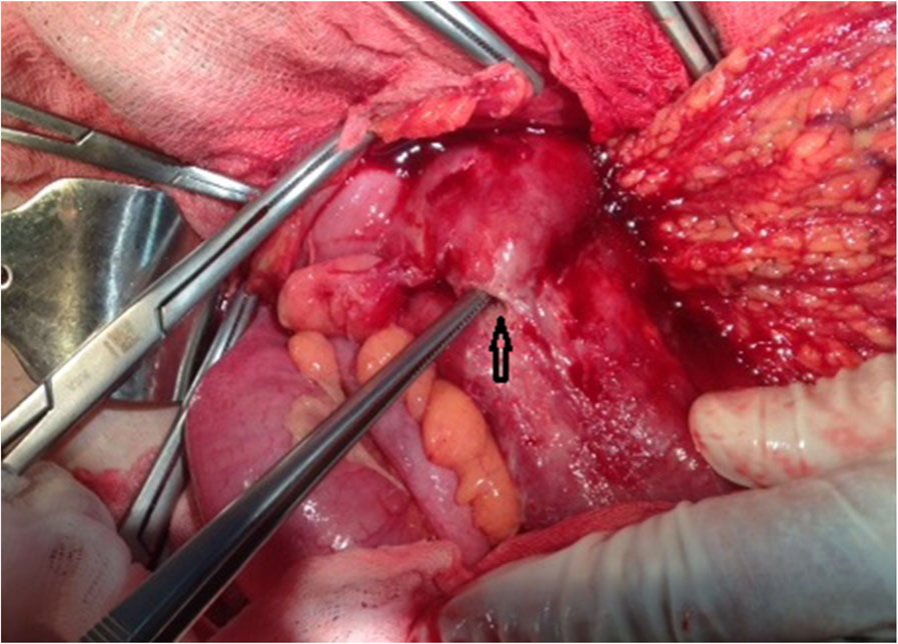
Introperative view of the terminal ileum perforation (*arrow*) with localized peritoneal and omental reaction

A 12-year-old child was admitted to our Unit after 2 weeks of persistent symptoms of headache, nausea and abdominal pain. Physical examination revealed stiff neck, left oculomotor paresis, left facial nerve paresis, left hemiparesis; laboratory tests showed a neutrophilic leukocytosis, thrombocytosis, increased C-reactive protein and a raised erythrocite sedimentation rate. Ophthalmological exam and fundus evaluation showed a bilateral papilledema and retinal vasculitis with loss of visual acuity. Brain computed tomography (CT) scan was performed and was normal. A brain magnetic resonance imaging (MRI) confirmed papilledema and showed a mild inversion of the optic papilla on the optic disk on both sides, with prominent subaracnoid space around the optic nerves, and a partially empty sella turcica. These findings appeared compatible with a condition of Pseudotumor cerebri (PTC) (Fig. 1). Therefore, a lumbar puncture was performed and revealed elevated cerebrospinal fluid pressure and oligoclonal bands of IgG, which were found also in the blood serum. Infective diseases were excluded. In the following days, abdominal pain and rectal bleeding appeared. Faecal calprotectin was increased, ANCA and ASCA autoantibodies were negative, HLA B51 was not found; a pathergy test was performed and the results were negative. Abdominal ultrasound revealed thickening of the ascending colon walls and cecal area, with a maximum thickness of about 6 mm with the loss of regular parietal stratification, and a subsequent abdominal CT documented wall thickening of the last ileal loop and the proximal of the blind, which is associated with edematous imbibition of surrounding fatty tissue. An endoscopic evaluation was performed: ileo-colonscopy showed round-shaped ulcers with active bleeding in the ileal region and esophagogastroduodenoscopy showed gastric aphtous lesions. In addiction, a history of recurrent oral minor aphtosis (3-4 episodes/year) was highlighted about two years before; then, according to ICBD and PEDBD criteria, the diagnosis of BD with intestinal and neurological involvement was suspected and three-day therapy with endovenous methylprednisolone (1 gr/die) was started, followed by oral corticosteroid and cyclophosphamide replaced after three months by mycophenolate mofetil. Clinical symptoms gradually improved with regression of gastrointestinal bleeding and complete remission of the neurological manifestations of the PTC. Histological findings of the ileal mucosa showed swollen vascular endothelial cells of capillary vessels and small blood vessels, fibrotic necrosis of small vessels and bleeding, diffuse perivascular lymphocytic in mucosa and submucosa suggestive of intestinal vasculitis (Fig. 2). Treatment with corticosteroids and mycophenolate mofetil was followed for several months with the disappearance of neurological and gastrointestinal symptoms. The steroids dose was gradually reduced, with subsequent relapsing of gastrointestinal symptoms. A magnetic resonance enterography was performed to evaluate the small-bowel and showed marked inflammatory changes of the terminal ileum and cecum with diffuse bowel-wall thickening and polypoid appearance. Localized fibrofatty proliferation and mesenteric vascular engorgement were also detected. Treatment with Adalimumab was started with transient improvement. After three months, the gastrointestinal symptoms relapsed again and the patient was submitted to urgent terminal-ileum resection for terminal ileal perforation (Fig. 3). Histological examination of the surgically resected intestinal specimens showed thickening of the vessel wall and infiltration of inflammatory cells (i.e. neutrophils and mononuclear cells) in the vascular wall and perivascular area, compatible with submucosal phlebitis pattern. No post-operative complications were observed, the therapy with adalimumab was suspended and maintenance therapy with azathioprine was started. No post-operative primary intestinal recurrence has appeared in the following 2 years, during which the patient didn’t presented oral aphtosis any more.

## Discussion

This clinical case demonstrates that BD is not a rare clinical entity in the paediatric population. Neurological involvement affects 5–10% of patients, is quite common in adults, and rarely occurs in children and adolescents [11, 12]. It involves the central nervous system, with parenchymal and non-parenchymal lesions, and rarely the peripheral nervous system. A parenchymal pattern includes focal parenchymal lesions and aseptic meningoencephalitis while non-parenchymal or vascular lesions include venous thrombosis and arterial vasculitis [13]. Our clinical case had symptoms of acute meningitis with stiff neck and headache, signs of peripheral nervous system involvement, such as left hemiparesis with palpebral ptosis, non-parenchymal findings of idiopathic intracranial hypertension (papilledema, visual impairment, headache) and ocular alterations (retinal vasculitis). In intestinal BD, the terminal ileum and cecum are the main sites involved and include volcano-type and aphthous type of lesions. Intestinal BD shares clinical course, endoscopic and histologic features with IBD, particularly Crohn’s disease (CD) [14–17]. In this case report, endoscopic evaluation showed round-shaped ulcers with active bleeding in the ileal region with gastric aphthous lesions. This macroscopic pattern can be differentiated from CD where longitudinal ulcers are discontinuous with mucosal edema, focal and diffuse erythema, nodular lesions, erosions and ulcers [18]. Neutrophilic infiltration, lymphocyte aggregation of the surrounding vessels, and vascular proliferation can be considered as histopathological characteristics of intestinal BD [19]. Ulcers in BD tend to perforate at multiple sites, with the need for surgery in up to 50% of patients [20]. Recurrent ulceration of the stoma is a relatively common complication, as the recurrence of disease adjacent to or at surgical anastomosis. Most of these recurrent ulcers appear within two years of resection [21].

## Conclusions

Although there is no specific diagnostic test for BD, diagnostic clinical criteria are available. Rheumatologists and gastroenterologists are mainly involved in the diagnosis and management of this disease. We believe that the report of this clinical case can be useful to paediatricians to make an appropriate differential diagnosis between BD and CD.

## Abbreviations

BD: Behçet’s disease
CD: Crohn disease
CT: Computed tomography
IBD: Inflammatory bowel diseases
ICBD: International Criteria for Behcet’s Disease
ISG: International Behcet’s Study Group
MRI: Magnetic resonance imaging
PEDBD: Paediatric BD
PTC: Pseudotumor cerebri

## References

[1] Sakane T, Takeno M, Suzuki N (1999). Behçet’s disease. N Engl J Med.

[2] Behçet H (1937). Rezidivierende aphthose, durch ein virus verusachte geschwure am auge und an den genitalien. Dermatol Wochenschr.

[3] Ciccarelli F, De Martinis M, Ginaldi L (2014). An update on autoinflammatory diseases. Curr Med Chem.

[4] Kalayciyan A, Zouboulis C (2007). An update on Behçet’s disease. J Eur Acad Dermatol Venereol.

[5] Caso F, Costa L, Rigante D (2014). Biological treatments in Behçet’s disease: beyond anti-TNF therapy. Mediators Inflamm.

[6] Koné-Paut I (2016). Behçet’s disease in children, an overview. Pediatr Rheumatol Online J.

[7] Koné-Paut I, Darce-Bello M, Shahram F (2011). Registries in rheumatological and musculoskeletal conditions. Paediatric Behçet’s disease: an international cohort study of 110 patients. One-year follow-up data. Rheumatology (Oxford).

[8] Gül A, Inanç M, Ocal L (2000). Familial aggregation of Behçet’s disease in Turkey. Ann Rheum Dis.

[9] Maldini C, Lavalley MP, Cheminant M (2012). Relationships of HLA-B51 or B5 genotype with Behcet’s disease clinical characteristics: systematic review and meta-analyses of observational studies. Rheumatology (Oxford).

[10] Koné-Paut I, Shahram F, Darce-Bello M (2016). Consensus classification criteria for paediatric Behçet’s disease from a prospective observational cohort: PEDBD. Ann Rheum Dis.

[11] Albakaye M, Louhab N, Chraa M (2014). Pseudotumoral form of neuro-Behçet in children. Rev. Neurol. (Paris).

[12] Dutra and Barsottini, Neuro-Behçet’s Disease: A Review of Neurological Manifestations and Its Treatment J Vasc 2016, 2:2 http://dx.doi.org/10.4172/2471-9544.100112

[13] Mora P, Menozzi C, Orsoni JG (2013). Neuro-Behçet's disease in childhood: a focus on the neuro-ophthalmological features. Orphanet J Rare Dis.

[14] Yazısız V (2014). Similarities and differences between Behçet's disease and Crohn's disease. World J Gastrointest Pathophysiol.

[15] Kim DH, Cheon JH (2016). Intestinal Behçet’s Disease: A True Inflammatory Bowel Disease or Merely an Intestinal Complication of Systemic Vasculitis?. Yonsei Med J.

[16] Grigg EL, Kane S, Katz S (2012). Mimicry and deception in inflammatory bowel disease and intestinal behcet disease. Gastroemterol Hepatol.

[17] Bayraktar Y, Ozaslan E, Van Thiel DH (2000). Gastrointestinal manifestations of Behcet’s disease. J Clin Gastroenterol.

[18] Annunziata ML, Caviglia R, Papparella LG, Cicala M (2012). Upper gastrointestinal involvement of Crohn’s Disease: a prospective study on the role of upper endoscopy in the diagnostic workup. Dig Dis Sci.

[19] Matsumoto T, Uekusa T, Fukuda Y (1991). Vasculo-Behçet’s dis- ease: a pathologic study of eight cases. Hum Pathol.

[20] Moon CM, Cheon JH, Shin JK (2010). Prediction of free bowel perforation in patients with intestinal Behçet’s disease using clinical and colonoscopic findings. Dig Dis Sci.

[21] Turan M, Sen M, Koyuncu A, Aydin C, Arici S (2003). Sigmoid colon perforation as an unusual complication of Behçet's syndrome: report of a case. Surg Today.

